# CBX2 Deletion Suppresses Growth and Metastasis of Colorectal Cancer by Mettl3-p38/ERK MAPK Signalling Pathway

**DOI:** 10.7150/jca.92633

**Published:** 2024-02-24

**Authors:** Rui Sun, Xucan Tu, Shixin Chan, Xu Wang, Yizhong Ji, Zhenglin Wang, Zhen Yu, Xiaomin Zuo, Qing Zhang, Jiajie Chen, Qijun Han, Ming Wang, Hu Zhao, Huabing Zhang, Wei Chen

**Affiliations:** 1 Department of General Surgery, First Affiliated Hospital of Anhui Medical University, Hefei 230032, China.; 2 Department of Biochemistry and Molecular Biology, Metabolic Disease Research Center, School of Basic Medicine, Anhui Medical University, Hefei 230032, China.; 3 Anhui Provincial Institute of Translational Medicine, Hefei 230032, China.; 4 Department of Dermatology, First Affiliated Hospital of Anhui Medical University, Hefei 230032, China.

**Keywords:** CBX2, METTL3, MAPK signal pathway, proliferation, metastasis, liver metastasis

## Abstract

Colorectal cancer (CRC) seriously endangers human health owing to its high morbidity and mortality. Previous studies have suggested that high expression of CBX2 may be associated with poor prognosis in CRC patients. However, its functional role in CRC remains to be elucidated. Herein, we found that CBX2 overexpression in colorectal cancer tissue compared with adjacent tissues. Additionally, forest maps and the nomogram model indicated that elevated CBX2 expression was an independent prognostic factor in CRC. Moreover, we confirmed that the deletion of CBX2 markedly suppressed the proliferation and migration of CRC cells *in vitro* and *in vivo*. Furthermore, downregulation of CBX2 promotes CRC cell apoptosis and hinders the cell cycle. Mechanistically, our data demonstrated that deletion of CBX2 inhibited the MAPK signaling pathway by regulating the protein levels of Mettl3. In conclusion, our study demonstrated that CBX2 is a vital tumor suppressor in CRC and could be a promising anti-cancer therapeutic target.

## Introduction

Colorectal cancer (CRC), one of the most widespread malignancies, is the second main global cancer-related cause of death.^1^ The initiation and progression of CRC are multistep and complex processes caused by multiple environmental, lifestyle, and genetic risk factors. However, the exact causes need to be further explored.^2^ Despite tremendous therapeutic and diagnostic advances in CRC, the effects of treatment have remained unsatisfactory over the past decades.^3^ Therefore, novel screening and therapeutic strategies for CRC are urgently needed.

Chromobox (CBX) members, such as CBX1-8 proteins, are essential components of polycomb group (PcG) protein complexes.^4^ Several members of the PcG complex have been described as epigenetic drivers in human cancers, including CRC.^5^ CBX2, an oncogene, has been associated with several tumours, including tumours originating from liver, stomach and prostate.^6^-^8^ Although recent research revealed that CBX2 expression and prognostic significance may be associated with CRC,^9^ more convincing evidence is still needed.

The mitogen-activated protein kinase (MAPK) is a classic cellular phosphorylation cascade that participates in a wide range of cellular and physiological processes essential to the life^10^ and has been considered one of the most frequently affected cancer signaling pathways.^11^,^12^ It consists of three classical branches: ERK, JNK, and p38 MAPK.^13^ Sun et al. demonstrated that activation of ERK promotes m6A methylation by phosphorylating Mettl3.^14^ Methyltransferase-like 3 (Mettl3), one of the main methyltransferases for the m6A methylation,^15^ has been reported that it promotes various tumours growth, including CRC.^16^,^17^ These results suggest a close relationship between MAPK pathway and Mettl3. In previous studies, it has been demonstrated that MAPK pathway activation promotes epithelial-mesenchymal transition (EMT) and the cell cycle in a variety of tumours, 18,19 including CRC.^20^-^22^

This study examined the function of CBX2 in CRC progression. CBX2 knockout (KO) CRC cell lines were constructed to study the role CBX2 plays in CRC occurrence and development using CRISPR/Cas9 system. Herein, we confirmed that CBX2 is involved in the growth and metastasis of CRC by controlling Mettl3-p38/ERK MAPK signaling pathway. This may reveal a significant target for therapeutic interventions in CRC.

## Materials and Methods

### Tissue collection

This study included 111 patients with CRC who underwent surgery without chemotherapy. From June 2014 to September 2016, the data was gathered at the Department of General Surgery, the First Affiliated Hospital of Anhui Medical University. Inclusion criteria: ① Patients met the criteria for clinical diagnosis of colorectal cancer and had indication for resection; ② Patients and their families were informed about the study and signed the relevant instruments. Exclusion criteria: ① Combination of serious heart, lung, liver and kidney diseases; ② Combination of other malignant tumors; ③ Recurrent tumors. Clinical and pathologic features, including sex, age, and tumour stage, were extracted from the medical records (Table S1).

### Immunohistochemistry and HE staining

The formalin-fixed tissues were sent to the Xinle Biological Company for embedding, sectioning, and staining with haematoxylin and eosin. Then antigen retrieval was performed using sodium citrate antigen retrieval solution (Solarbio, China). A universal two-step assay kit (pv-9000, ZSGB-BIO, China) and CBX2 antibody (Proteintech, China) were used to incubate the tissue sections. Finally, the antibody complexes were detected with DAB, and the sections were counterstained with haematoxylin. We used the IHC Profiler plug-in in ImageJ^23^ to obtain an IHC score which integrated staining percentage and staining intensity defined as 1: negative, 2: weak positive, and 3: positive. Accordingly, the low expression group for CBX2 was defined as an IHC score of 1, whereas the others were classified into the high group.

### Data mining

According to the IHC staining results, we divided the enrolled patients into low CBX2 expression group and high CBX2 expression group. Disease-free survival (DFS) analysis was conducted by Kaplan-Meier (KM) survival and log-rank test. Receiver operating characteristic (ROC) analysis was conducted by R package “survival ROC”. The Cox regression results were utilized to create a nomogram model for predicting the DFS of patients, and survival rates predicted by the nomogram were compared to the actual survival rates using calibration plots, and decision curve analysis (DCA) was utilzed to test the clinical applicability of the constructed nomogram model. The Shinyapps webserver via R studio software was used to build an online version of the nomogram model.

### Cell culture

The American Type Culture Collection was the source of all cell lines used in this investigation which treated with mycoplasma inhibitor SaveIt (HenBi, China) for one week every 3 months. Dulbecco's modified Eagle medium (DMEM) with 10% fetal bovine serum (FBS; Lonsera, Austria) was used as the culture medium. Under 5% CO2 at 37 °C, the cells were incubated.

### Cell transfection

The plasmids used are displayed in Table S3. For transfection of these plasmids, cells were grown to 80% confluence and were transfected using JetPRIME® (Polyplus-transfection S.A, France) based on the protocol of the manufacturer.

### Generation of CBX2 KO cell lines

CRC cell lines (HCT116 and HT29) were enome-edited using CRISPR/Cas9 vector, LentiCRISPRv2 (Genepharma, China), with a particular CBX2 guide RNA sequence (1st gRNA: 5'ACCAGCCGCGCCACTTGACC-3', 2nd gRNA: 5'CCTTGCGGAGCCGCTTGCTC-3'). 72 hours after transfection, single cells were arranged into 96-well plates by cell counting to obtain the CBX2 KO clones. CBX2 KO efficiency was confirmed by WB.

### RNA isolation and quantitative real‐time polymerase chain reaction (qPCR)

Total RNAs were extracted from the cell lines using the TRIzol (Life, USA) and qPCR was carried out by RevertAid First Strand cDNA Synthesis Kit (Thermo Fisher Scientific, USA), qPCR Master Mix (Universal, China) and a Roche LightCycler® 480II PCR machine based on the protocol of the manufacturer. Primers used are listed in Table S2.

### RNA-sequencing (RNA-seq) and analyses

Transcriptome sequencing and analyses were conducted using Biomarker Technologies Inc. software (Beijing, China). Briefly, purified RNA from KO and control cells was utilized for library construction using NEBNext® Ultra™ RNA Library Prep Kit (NEB, USA) and then sequenced using Novaseq6000. The HISAT2 was used to align raw data to the human genome GRCh38_release95. DESeq2 or edgeR were used to filter the differentially expressed genes (DEGs) between the KO and control groups, depending on the following criteria: false discovery rate (FDR) < 0.01, |log2FC| > 1. Sequencing data were deposited to NCBI under SRA accession numbers (SRA: PRJNA858965).

### Western blot (WB) analysis

WB analysis was performed as previously described.^24^ The primary antibodies were employed: CBX2 (Proteintech, China), CDK4/6 (Proteintech, China), cyclin D1 (Proteintech, China), Flag (Sigma-Aldrich, USA), Ha (Abmart, China), Mettl3 (Abclonal, China), and GAPDH (Abcam, England).

### Immunoprecipitation (IP)

In total, 293 T cells transfected with plasmids were taken using a scraper and lysed in cell lysis buffer (Beyotime). Then they were pre-cleared and immunoprecipitated with anti-FLAG or anti-Ha magnetic beads (Bimake). At last, the IP product was subjected to WB analysis.

### Flow Cytometry

Apoptosis and cell cycle distribution analyses were carried out on a FACSCalesta flow cytometer (BD Biosciences). HCT116 and HT29 cells were incubated until it reached 85% confluency before harvesting and analysis. The processing of cells was based on the guidelines of Annexin V-FITC/PI Apoptosis Detection Kit and Cell Cycle Assay Kit (BestBio, China).

### Cell proliferation and migration assay

MTT, colony formation and wound healing assays were performed as previously described.^24^ For matrigel invasion assay, transwell chambers (Corning, USA) covered with Matrigel (Corning, USA) were used for another migration experiment. Cells (8×10^4^) were positioned in the top chamber under conditions with no serum when a medium containing 10% fetal bovine serum was used in the bottom chambers. After 2 days, we scrubbed inner chambers and cells which had moved to the other side of the membrane were exposed to fixing, staining with crystal violet and counting under a microscope.

### Animal studies

Male BALB/c mice were brought from Gempharmatech Limited Company and kept in specific facilities with no pathogen at Anhui Medical University. For the nude mouse tumour formation assay, it was performed as previously described.^24^ For an animal model of liver metastasis, the suspension (5×10^6^ cells in 200 μl PBS) was given by injection into the lower pole of the mouse spleen after an open operation and mice underwent sacrifice after five weeks. The liver and lungs were taken for follow-up studies.

### Immunofluorescence

After antigen retrieval, sections were treated with 5% goat serum (ZSGB-BIO, China) for 30 min, and then the primary antibody (1:100 dilution), such as Ki67 and cleaved caspase 3 (ABclonal, China), was added drop by drop and inserted into the incubator overnight. After incubation with fluorescein isothiocyanate (FITC) secondary antibody and 4',6-diamidino-2-phenylindole (DAPI), the sections were immediately mounted and observed with a fluorescence microscope.

## Results

### CBX2 expression is upregulated in CRC tissue samples

**Figure 1:**
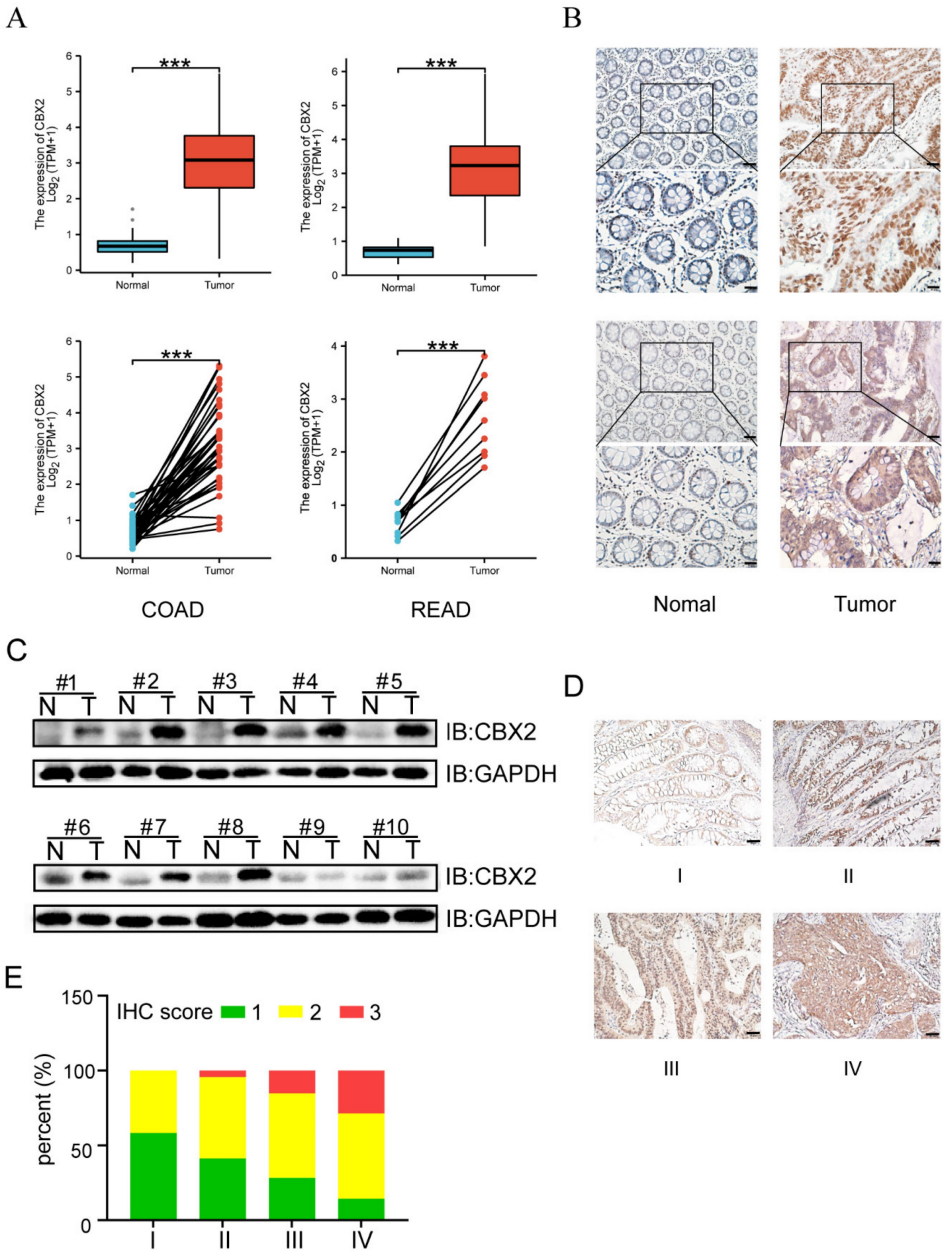
** CBX2 expression is up-regulated in human CRC tissues.** (A) TCGA database shows the relative RNA expression level of CBX2 in healthy and CRC patients. (B) IHC analysis shows the CBX2 protein expression levels in CRC (Tumour) or the paired non-tumour tissues (Normal), (Scale bar: 50μm and 100μm). (C) WB analysis shows the CBX2 protein expression levels in CRC or the paired non-tumour tissues. (D) CBX2 expression in CRC tissues at stage I (n=12), stage II (n=46), stage III (n=46) and stage IV (n=7) by IHC staining (Scale bar: 50μm). (E) The results of IHC score quantification in different stages. Data are shown as mean ± SD; ***P < 0.001.

Expression of CBX2 was shown to be significantly higher in CRC tissues than in paracancerous tissues (Figure 1A). Moreover, IHC and WB further established that CBX2 protein level was noticeably increased in colorectal neoplasia tissues (Figure 1B and C). Additionally, with the increase of tumour staging, the expression level of CBX2 also increased significantly (Figures 1D and E), suggesting that CBX2 may be related to a low prognosis in patients with CRC.

### Depletion of CBX2 inhibits cell proliferation and arrests cell cycle

**Figure 2:**
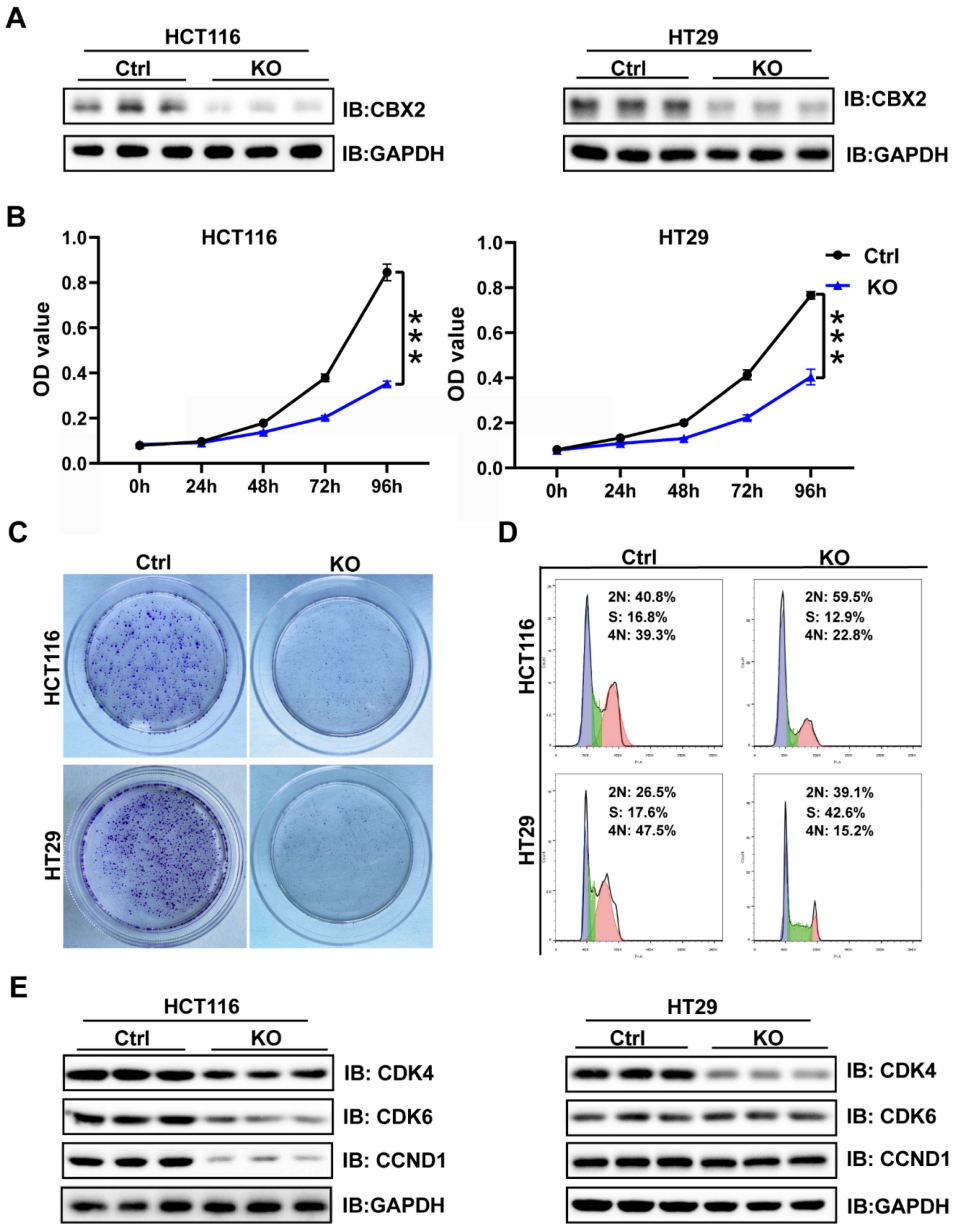
** Depletion of CBX2 inhibits CRC cells proliferation and arrests cell cycle.** (A) WB is used to assess the KO efficiency in HCT116 and HT29 cells. (B, C) MTT (B) and colony formation assay (C) are performed to assess cell proliferation. (D) Flow cytometry analysis is used to detect cell cycle distribution. (E) WB is used to assess the alteration of cell cycle-related proteins (CDK4, CDK6 and CCND1). Data are shown as mean ± SD; ***P < 0.001.

WB and qPCR were conducted to analyse CBX2 expression level in five CRC cell lines (HCT116, HT29, SW480, RKO, and LOVO) and a normal intestinal epithelial cell line (NCM460). The results presented that CBX2 expression showed significant increase in CRC cell lines than in normal cell lines (Figure S1A and B). Then we used the CRISPR/Cas9 system to delete CBX2 expression in two different cell lines (HCT116 and HT29). WB results showed that CBX2 expression levels in KO cell lines was significantly decreased compared to the control cells (Figure 2A). MTT and clone formation assay identified that ablation of CBX2 inhibited cell proliferation (Figures 2B and C). Although flow cytometry analysis revealed that CBX2 KO had no significant effect on apoptosis (Figure S2A), we discovered that G1/S phase cells elevated after deletion of CBX2, while G2 phase cells significantly reduced (Figure 2D). We also analyzed the expression of genes associated with the cell cycle using WB. As expected, cyclin-dependent kinase 6 (CDK6) and cyclin D1 (CCND1) were significantly decreased in CBX2 KO cells in comparison with the control cells (Figure 2E).

### Ablation of CBX2 inhibits CRC cells migration and invasion

**Figure 3:**
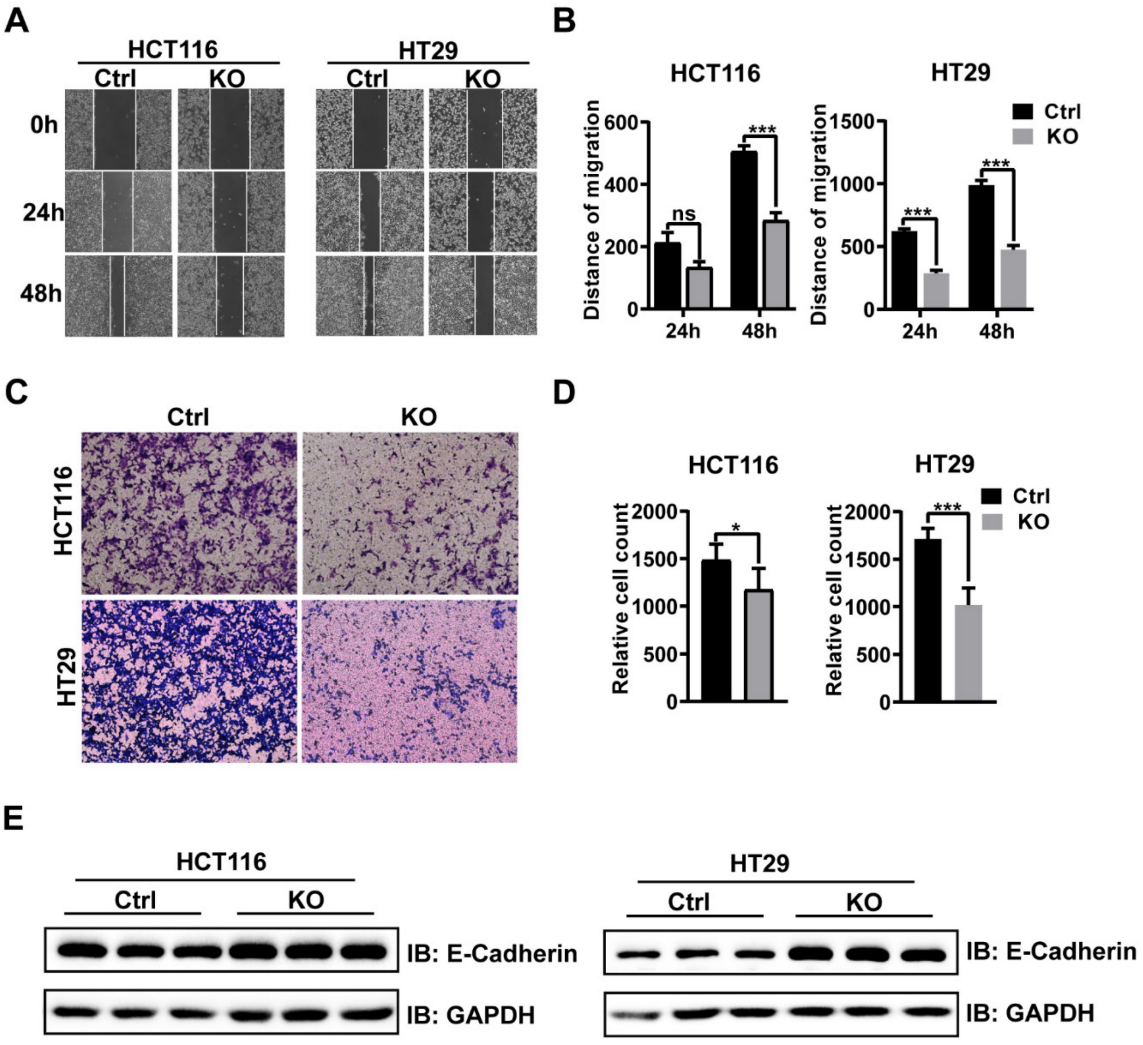
** CBX2 dwficiency inhibits CRC cell migration and invasion.** (A, B) Wound healing assay is used to evaluate the migration. (C, D) Transwell assay is utilized to evaluate the invasion. (E) WB analysis of EMT markers in HCT116 and HT29 cells. Data are shown as mean ± SD; *P < 0.05, ***P < 0.001.

The results of wound healing assay confirmed that CBX2 KO inhibited cell migration in both the cell lines (Figures 3A and B). Furthermore, HCT116 and HT29 cells invasion capabilities were also impaired after CBX2 deletion (Figures 3C and D). Overall, our results demonstrate that depletion of CBX2 suppressed the aggressiveness of CRC cells. Moreover, the expression of epithelial-mesenchymal transition (EMT) markers, such as E-cadherin, was noticeably reduced in CBX2 deletion HCT116 and HT29 cell lines (Figure 3E). These data indicate that deletion of CBX2 suppresses CRC cells migration and invasion via regulating the EMT pathway.

### Transcriptome profiling analysis reveals CBX2 deletion suppressed the MAPK signalling pathway

**Figure 4:**
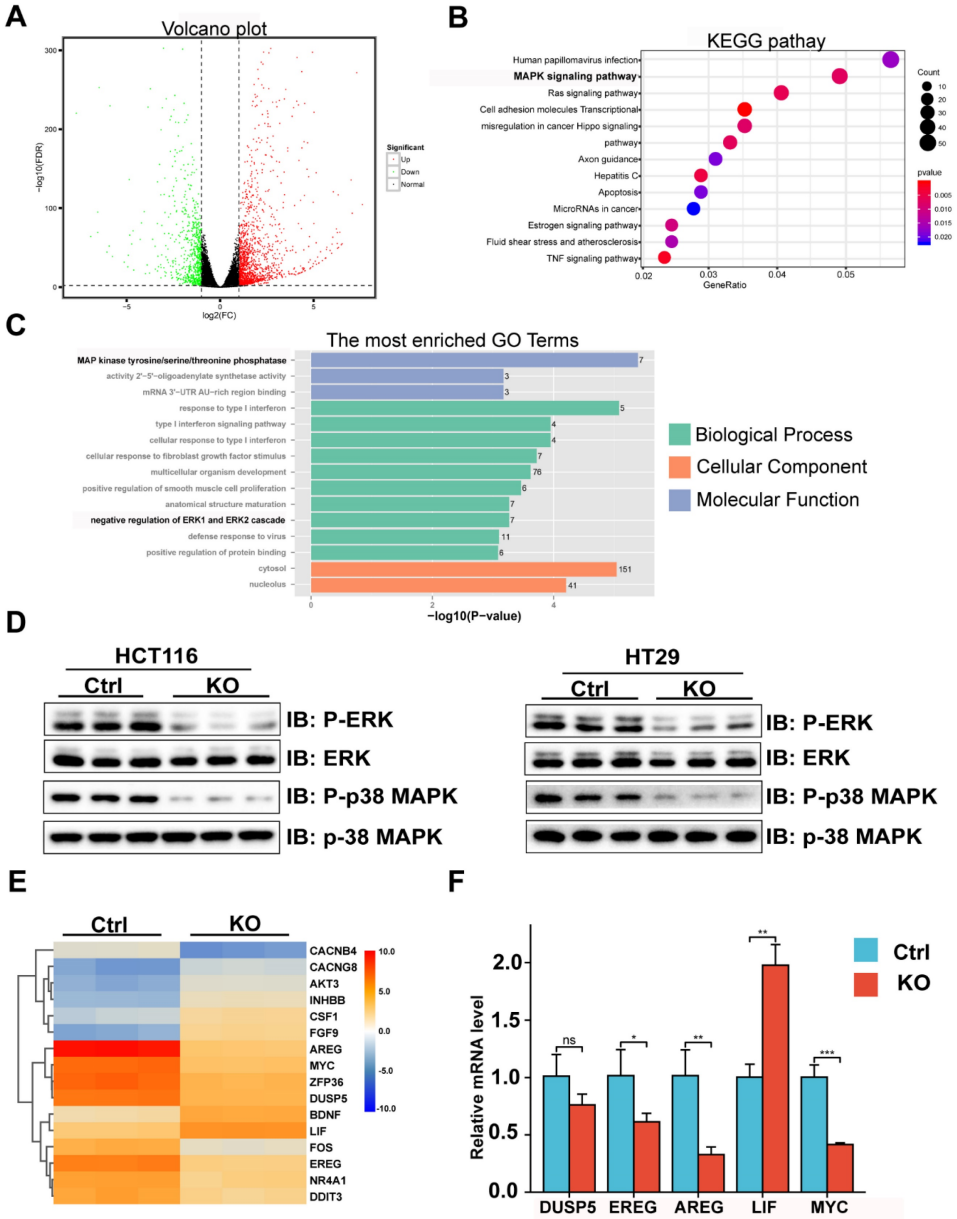
** Transcriptome profiling analysis indicates CBX2 deletion suppressed the MAPK signalling pathway.** (A) Scatterplot of differentially expressed genes of RNA-seq in CBX2 knockout HCT116 cells and control cells. (B) KEGG pathway analysis showing enriched pathways by RNA-seq. (C) GO pathway analysis showing enriched pathways by RNA-seq. (D) WB analysis showed that deletion of CBX2 decreased the phosphorylation levels of ERK and p38 MAPK. (E) The heatmap about differentially expressed genes in the MAPK pathway is present. (F) qPCR is used to confirm the results of the heatmap. Data are shown as mean ± SD; *P < 0.05, **P < 0.01, ***P < 0.001.

We further explored the potential mechanism of CBX2 affecting CRC cell proliferation and migration. Using transcriptome analysis, a total of 2284 DEGs were screened by DESeq2 or edgeR and are displayed in the volcano plot (Figure 4A). Then, we discovered that MAPK signalling pathway was enriched by using Kyoto encyclopedia of genes and genomes (KEGG) analysis (Figure 4B). Moreover, gene ontology (GO) (Figure 4C) analysis also suggested that deletion of CBX2 led to phosphorylation change of MAP kinase in biological process and regulation of ERK cascade in molecular function. Furthermore, WB analysis confirmed that the phosphorylation of ERK and p38 MAPK showed significant decrease in the CBX2 KO group (Figure 4D). Lastly a heatmap (Figure 4E) showed that 16 genes related to the MAPK signalling pathway are differently expressed in CBX2 KO group and control group, which have been validated by qPCR (Figure 4F).

### CBX2 regulates MAPK signalling pathway from stabilizing Mettl3 expression

**Figure 5:**
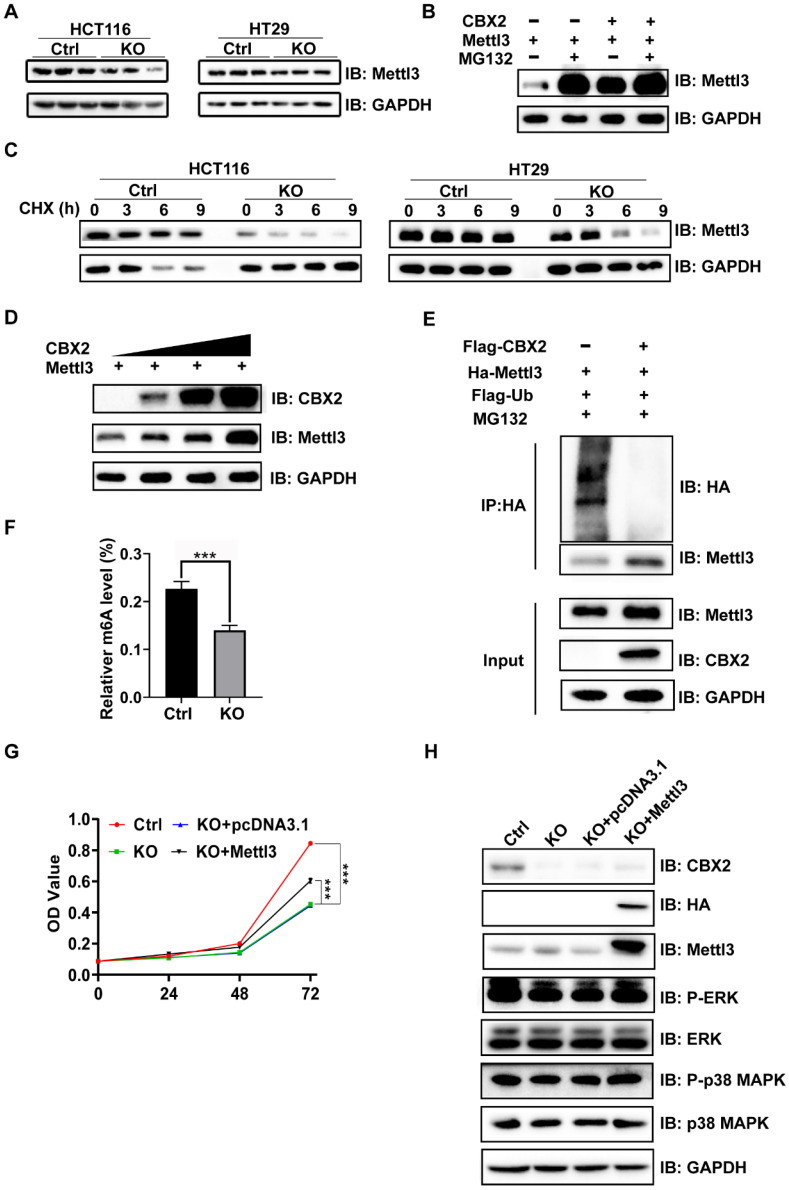
** CBX2 regulates MAPK signalling pathway by stabilizing the expression of Mettl3.** (A) Mettl3 expression level in HCT116 and HT29 cells with or without CBX2 KO. (B) WB of indicated proteins in 293A cells treated with or without MG132 (6 hours, 20μmol/L) after being transfected plasmid Mettl3, CBX2 or pc-DNA3.1. (C) WB of indicated proteins in HCT116 and HT29 cells with or without CBX2 KO treated with CHX (20 μg/ml). (D) WB of indicated proteins in 293A cells after being transfected plasmid Mettl3 and CBX2. (E) The IP assay is performed to detect whether CBX2 inhabits the ubiquitination of Mettl3 in 293A cells. (F) The m6A level of HCT116 cells is detected by EpiQuik m6A RNA Methylation Quantification Kit. (G) MTT assay is utilized to evaluate the proliferation of the indicated cells. (H) WB of indicated proteins in HCT116 CBX2 KO cells after being transfected plasmid CBX2 and pc-DNA3.1. Data are shown as mean ± SD; ***P < 0.001.

Previous studies have reported that the phosphorylation level of MAPK may be regulated by Mettl3.^14^,^25^-^27^ Furthermore, it has been reported that Mettl3 expression level is drastically upregulated in CRC tissues in comparison with normal tissues, and knockdown of Mettl3 reduces the phosphorylation levels of ERK and p38 MAPK and inhibits CRC tumourigenesis and metastasis.^16^ Notably, our results confirmed that CBX2 KO in CRC cells caused a significant reduction in Mettl3 protein expression (Figure 5A), which could be rescued by the proteasome inhibitor MG132 (Figure 5B). As showed in Figure 5B, either CBX2 or MG132 increased the expression level of Mettl3, but when MG132 was added, whether CBX2 was added or not had no impact on Mettl3 expression. These findings indicated that CBX2 may regulate Mettl3 expression via the proteasome pathway. Furthermore, Cycloheximide (CHX), an inhibitor of protein synthesis, decreased Mettl3 protein concentrations more quickly in CBX2 deficient CRC cells than in control cells (Figure 5C). Moreover, we discovered that there was a positive relationship between CBX2 and Mettl3 expression levels (Figure 5D). Furthermore, the IP assay identified that upregulation of CBX2 decreased the ubiquitination level of Mettl3 (Figure 5E). However, we found no direct interaction between CBX2 and Mettl3 (Figure S2B). Numerous studies have confirmed that Mettl3 is the major methyltransferase responsible for m6A modification,^28^ so we compared m6A levels between the KO group and control group. Our data identified that significantly decreased levels of m6A were found in the KO group compared to the control group. (Figure 5F). To further validate this, we overexpressed Mettl3 in HCT116 KO cells using the plasmid pcDNA3.1-Mettl3-Ha, and the MTT assay established that over-expression of Mettl3 restored CBX2 KO inhibitory effect (Figure 5G). It has been stated that elevation of Mettl3 expression can elevate ERK and p38 MAPK phosphorylation.^29^ We also found that upregulated Mettl3 expression restored ERK and p38 MAPK phosphorylation to some extent in CBX2 KO CRC cells (Figure 5H). These findings show CBX2 regulates MAPK signalling pathway by stabilizing Mettl3 expression.

### CBX2 inactivation inhibits CRC cells proliferation and metastasis in vivo

**Figure 6:**
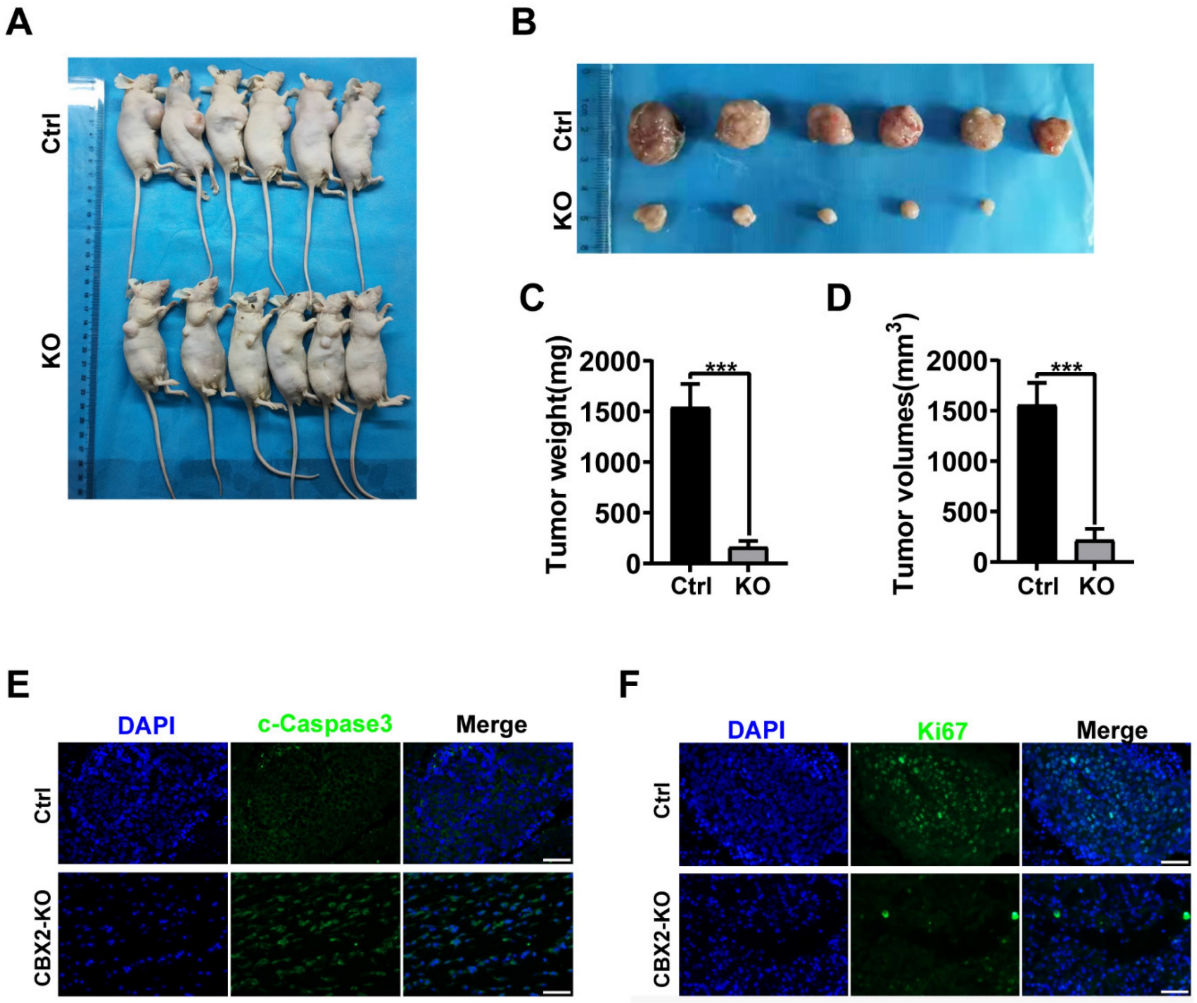
** CBX2 inactivation inhibits cell proliferation *in vivo.*** (A, B) Control and CBX2 deletion HCT116 cells are subcutaneously injected into BALB/c nude mice to observe the tumour growth (n=6/group). (C, D) The tumour weight and volume. (E, F) Immunofluorescence staining for Ki67 (E) or cleaved-Caspase3 (F) in tumour tissues from nude mice (Scale bar: 50μm). Data are shown as mean ± SD; **P < 0.01, ***P < 0.001.

**Figure 7:**
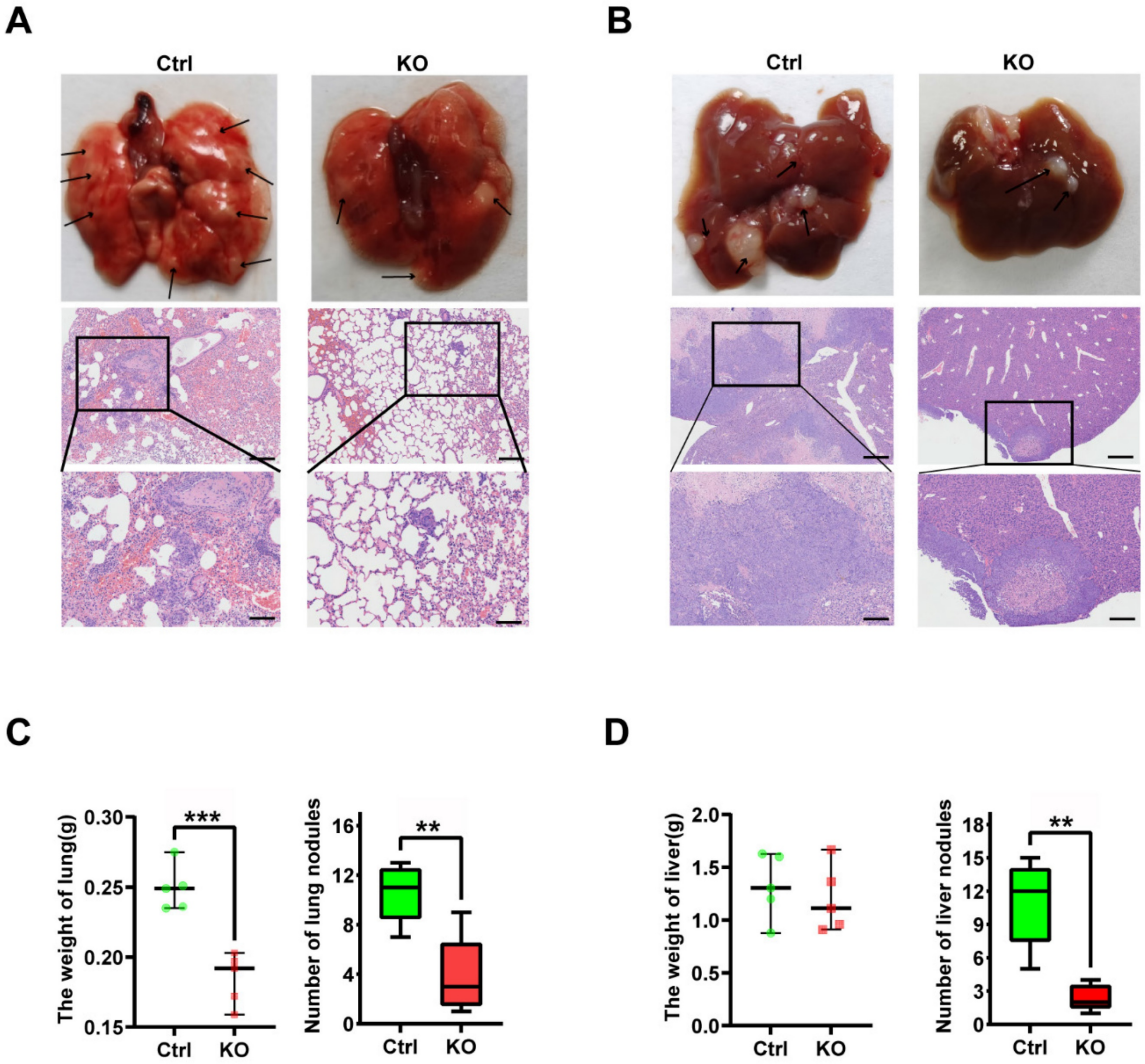
** CBX2 KO suppresses CRC cells metastasis *in vivo.*** (A, B) Control and CBX2 deletion HCT116 cells are injected into the spleen of BALB/c nude mice to observe the metastatic nodule in the lung (A) and liver (B), and HE staining for the lung (Scale bar: 100μm and 200μm) and liver (Scale bar: 200μm and 500μm) tissue. (C, D) The lung (C) or liver (D) weight and metastatic nodules. Data are shown as mean ± SD; **P < 0.01, ***P < 0.001.

To further explore whether CBX2 affects tumourigenesis *in vivo*. Xenograft tumours were generated in nude mice by injecting CBX2 deletion HCT116 and control cells subcutaneously and examined tumour growth. We found that deletion of CBX2 markedly suppressed tumour growth and reduced tumour size (Figures 6A and B), volume (Figures 6C), and weight (Figure 6D) in comparison with the control group. The immunofluorescence analysis revealed a lower percentage of Ki-67-positive cells and a higher level of cleaved-caspase3-positive cells in tumours derived from CBX2 KO cells than in tumours derived from control cells (Figures 6E and F). Furthermore, we constructed lung and liver metastasis models by injecting CBX2 deletion HCT116 cells or control cells into the spleens of mice after laparotomy for 8 weeks. The results showed that CBX2 KO decreased the count of metastatic pulmonary and liver nodules compared to the control groups (Figures 7A and B). Meanwhile, the CBX2 KO group had lower lung weight than the control group (Figure 7C). Although liver weight showed no difference between two groups, the difference of hepatic nodule number has statistical significance (Figure 7D). These data suggest that deletion of CBX2 significantly suppresses growth and migration of CRC cells *in vivo*.

### Upregulation of CBX2 has a correlation with low prognosis in CRC patients

**Figure 8:**
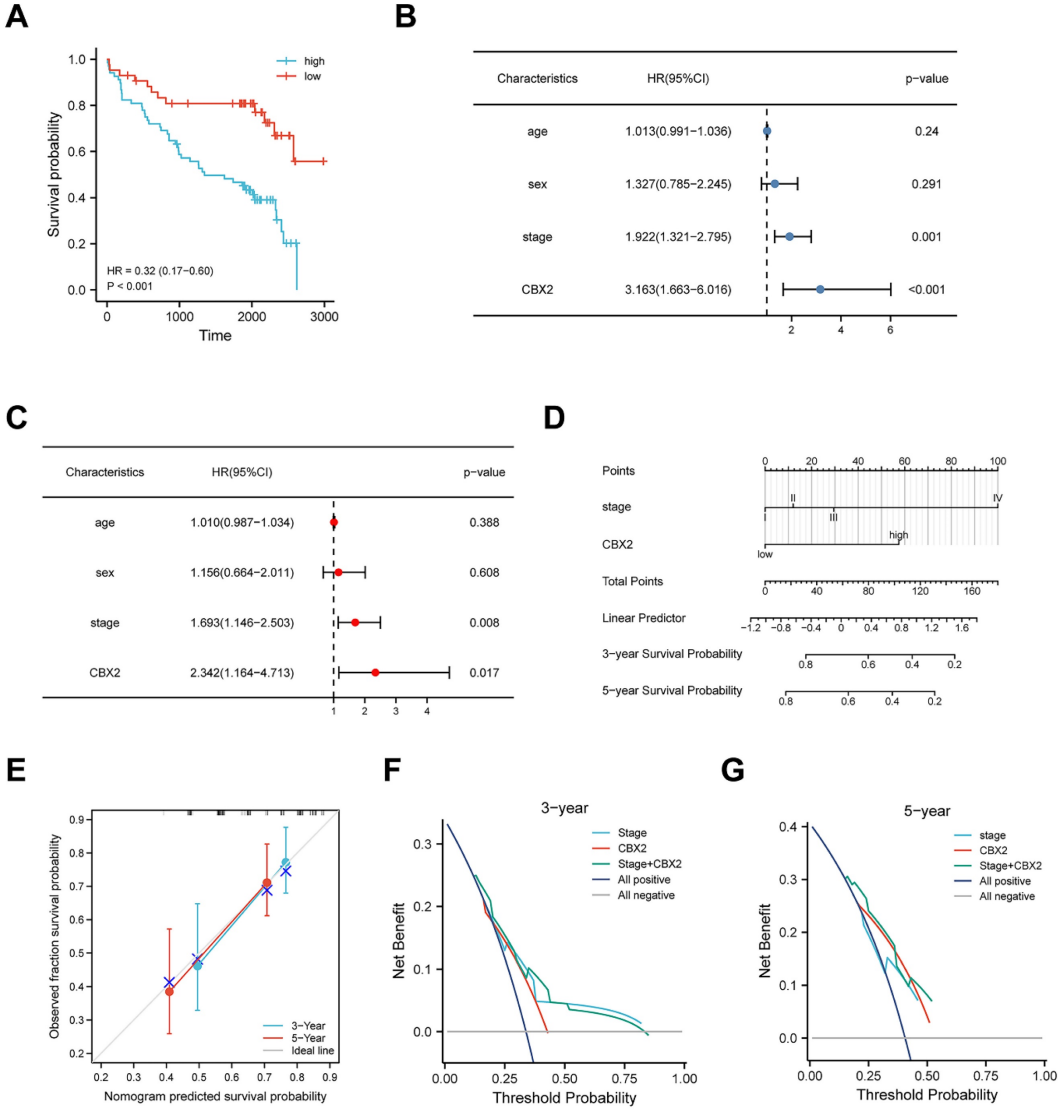
** High expression of CBX2 is associated with poor prognosis in CRC patient tissue.** (A) Patients with high CBX2 expression have longer disease-free survival (DFS) time tested by Kaplan-Meier test. (B, C) Forest maps generated from Univariate (B) and Multivariate (C) Cox regression analyse show CBX2 is a prognosis-related factor. (D) A nomogram model is constructed to predict the DFS of CRC patients. (E) The calibration plots show the nomogram has great predictive power. (F, G) DCA shows the clinical practicability of the nomogram model.

Figure 8A shows the KM curve for DFS differences between low-CBX2 and high-CBX2 groups in CRC patients. Patients with CBX2 overexpressed had a significantly poorer DFS probability (p < 0.001). Univariate and multivariate Cox regression were used to evaluate prognostic factors. Forest maps (Figures 8B and C) showed that American Joint Committee on Cancer (AJCC) stage and CBX2 expression remained significant after univariate and multivariate analyses, indicating that these two factors could independently predict patient DFS. As per Cox regression analysis results, AJCC stage, and CBX2 expression were included to construct the nomogram model (Figure 8D). Calibration plots showed the predicted 3- and 5-year DFS rates were close to the actual DFS rates (Figure 8E), indicating that the model reliably and accurately predicted DFS in CRC patients. Three- (Figure 8F) and five-year (Figure 8G) DCA curves indicated that the constructed nomogram model had better clinical applicability than other clinical features because of the higher net benefit with wide ranges of threshold probabilities. The online version of the constructed nomogram model was built to provide accurate and individualised survival predictions for patients with CRC (https://https://doctorwang.shinyapps.io/CBX2/). Using this model, accurate survival rates can be easily calculated by inputtingcompare patients' clinical variables.

## Discussion

In this study, we discovered that elevated CBX2 expression was positively related to the malignancy of colorectal cancer, as well as provided new insights into its functional role in promoting CRC proliferation and migration.

CBX2, an important regulator of tumour progression and cell cycle,^30^ has been widely identified to be a tumour-promoting gene.^31^,^32^ Our results further confirmed that compared to non-tumor tissues, CRC tissues had significantly greater levels of the CBX2 protein. As expected, deletion of CBX2 significantly hampered cell proliferation and tumour progression *in vitro* and *in vivo*. A previous report confirmed that CBX2 knockdown can significantly suppress the proliferation and enhance apoptosis of hepatocellular carcinoma (HCC) cells* in vivo* and* in vitro*.^6^ However, our investigation presented that CBX2 deficiency promoted CRC cell apoptosis *in vivo* but not *in vitro*. This may be due to changes in the cellular microenvironment. For example, Chen et al. reported that crosstalk between cancer cells and tumour-associated macrophages is required for CRC metastasis.^33^ To elucidated the underlying mechanism by which CBX2 utilizes its function in CRC. We performed RNA-seq and found that the deletion of CBX2 inhibited the MAPK pathway. WB results confirmed that results. Earlier studies have presented that p38 MAPK has a function in the positive regulation of metastasis and invasion^34^,^35^ and p38 activity is required to maintain CRC cell metabolism.^36^ Moreover, a recent study reported that CBX2 could shape chromatin accessibility via the MAPK pathway in the acute myeloid leukaemia.^37^ Therefore, we believe that deletion of CBX2 influences the proliferation of colorectal cancer cells by affecting MAPK signalling pathway.

Several studies have reported that Mettl3 regulates phosphorylation of ERK and p38 MAPK.^29^,^38^ However, it is unclear how CBX2 regulates ERK and p38 MAPK activity. In this study, we thought Mettl3 is an oncogene in CRC. Firstly, the article reproted that Mettl3 was overexpressed in CRC tissues,^16^ and we discovered that Mettl3 expression showed significant reduction in CRC cells with CBX2 deletion compared to that in the controls. Furthermore, the proliferation of CBX2 KO cells were rescued by overexpression of Mettl3. Although the Mettl3 protein expression was significantly reduced, we did not identify significant alterations in Mettl3 mRNA levels in two groups. In fact, the protein degradation pathway, such as the ubiquitin-proteasome system (UPS) and the autophagy-lysosome pathway, was considered to elucidate this phenomenon.^39^ We confirmed Mettl3 degradation via UPS with the proteasome inhibitor MG132. A recent study reported that Mettl3 is subject to both ubiquitination and SUMOylation modifications.^40^ However, SUMOylation of Mettl3 does not affect its stability or location.^40^ Therefore, we focused on ubiquitination given the alteration in the Mettl3 protein level. The results of IP assay demonstrated that CBX2 stabilises Mettl3 through the ubiquitin-proteasome pathway, and co-expression of CBX2 and Mettl3 significantly reduced the ubiquitination level of Mettl3. Furthermore, we established that upregulation of Mettl3 expression in CRC cells with CBX2 deletion increased ERK and p38 MAPK phosphorylation levels. As a result, we speculated that CBX2 might affect the MAPK signalling pathway by regulating Mettl3.

Growing evidence has demonstrated that CBX2 is a chromatin modifier,^41^ whose function is usually at the RNA or DNA level. However, our study suggests that CBX2 regulates protein expression through the ubiquitin-proteasome pathway. This may become a new direction of CBX2 mechanism research. This study also has several limitations. First, it is regretful that this topic involves a long study period due to the collection of clinical samples and patient prognostic information (e.g., disease-free survival beyond 5 years). During this period, a study discovered that downregulation of CBX2 inhibits the aggressiveness of colorectal cancer cells.^9^ However, it is also the study of Zhou et al that demonstrates, on the other hand, the reliability of some of the cellular assay results in this study. What's more, we demonstrated that deletion of CBX2 inhibited the MAPK signaling pathway by regulating the protein levels of Mettl3. We further proposed that CBX2 exerts its pro-carcinogenic effects by regulating the ubiquitination degradation of Mettl3 altering the phosphorylation levels of ERK and P38. Secondly upregulated expression of CBX2 in CRC cell lines had no significant effect on cell proliferation and metastasis. A possible reason for this is that CBX2 background expression is very high in CRC cell lines. Even if the expression of CBX2 is increased, it may have little effect. We therefore intend to continue to explore the effects of CBX2 on colorectal cancer in CBX2 knockout mice and transgenic mice. Lastly, although we found that CBX2 could inhibit the ubiquitination degradation of Mettl3 and thus affect the phosphorylation of MAPK signaling pathway, co-immunoprecipitation assays did not find a direct interaction between CBX2 and Mettl3. We will do *in vitro* ubiquitination assay to further explore the relationship between CBX2 and Mettl3 in the future.

Taken together, our results indicate that CBX2 could be an important oncogene-related prognostic marker in CRC. We highlight the functional importance of CBX2 in mediating CRC progression by regulating the Mettl3/MAPK signalling pathway. Therefore, we believe that inhibition of CBX2 may be an efficient target for CRC treatment.

## Supplementary Material

Supplementary figures and tables.
