# Supplementary material for: CBX2 Deletion Suppresses Growth and Metastasis of Colorectal Cancer by Mettl3-p38/ERK MAPK Signalling Pathway
Source: J Cancer. 2024 Feb 24;15(8):2123–36. doi: 10.7150/jca.92633 (PMC10937286; doi:10.7150/jca.92633)
Supplement: Supplementary file 1 — Supplementary figures and tables. [file jcav15p2123s1.pdf]

Supplementary Material:

Table S1: Association between clinicopathologic features in CRC patients and CBX2 expression;

Characteristic	Low CBX2	High CBX2	p
n	43	68	
Age, n (%)			0.777
<=65	31 (27.9%)	46 (41.4%)	
>65	12 (10.8%)	22 (19.8%)	
Sex, n (%)			0.057
Female	14 (12.6%)	36 (32.4%)	
Male	29 (26.1%)	32 (28.8%)	
AJCC Stage, n (%)			0.085
I/II	27 (24.3%)	30 (27%)	
III/IV	16 (14.4%)	38 (34.2%)	
Survival Status, n (%)			< 0.001
Alive	31 (27.9%)	23 (20.7%)	
Recurrence	12 (10.8%)	45 (40.5%)	

Table S2: qPCR primer

qPCR primer	Forward primer	Reverse primer
36B4(human,mouse)	ATCCCTGACGCACCGCCGTGA	TGCATCTGCTTGGAGCCCACGTT
CBX2(human)	GCCCAGCACTGGACAGAAC	CACTGTGACGGTGATGAGGTT
Mettl3(human)	CCCTATGGGACCCTGACAGA	TGACACCAACCAAGCAGTGT
MYC(human)	CGGGTAGTGGAAAACCAGCCT	TCCAGATATCCTCGCTGGGC
DUSP5(human)	AGGGTGCCTACTGCACATTC	CTACCCTGAGGTCCGTCTGA
EREG(human)	GTGATTCCATCATGTATCCCAGG	GCCATTCATGTCAGAGCTACACT
AREG(human)	GTGGTGCTGTCGCTCTTGATA	CCCCAGAAAATGGTTCACGCT
LIF(human)	CCAACGTGACGGACTTCCC	TACACGACTATGCGGTACAGC
FOS(human)	CCGGGGATAGCCTCTCTTACT	CCAGGTCCGTGCAGAAGTC

Table S3: Plasmid

pcDNA3.1- Puro	Youbio Biological Technology Co., Ltd.<br>China
pcDNA3.1-Mettl3-HA	Youbio Biological Technology Co., Ltd.<br>China
pcDNA3.1-CBX2-Flag	Youbio Biological Technology Co., Ltd.<br>China
pcDNA3.1-Ub-Flag	Youbio Biological Technology Co., Ltd.<br>China
LentiCRISPRv2	GenePharma<br>China
lentiGuide-Puro	GenePharma<br>China
pVSVg	GenePharma<br>China
psPAX2	GenePharma<br>China

Figures:

Figure S1: Effect of overexpression of CBX2 on colorectal cancer cells

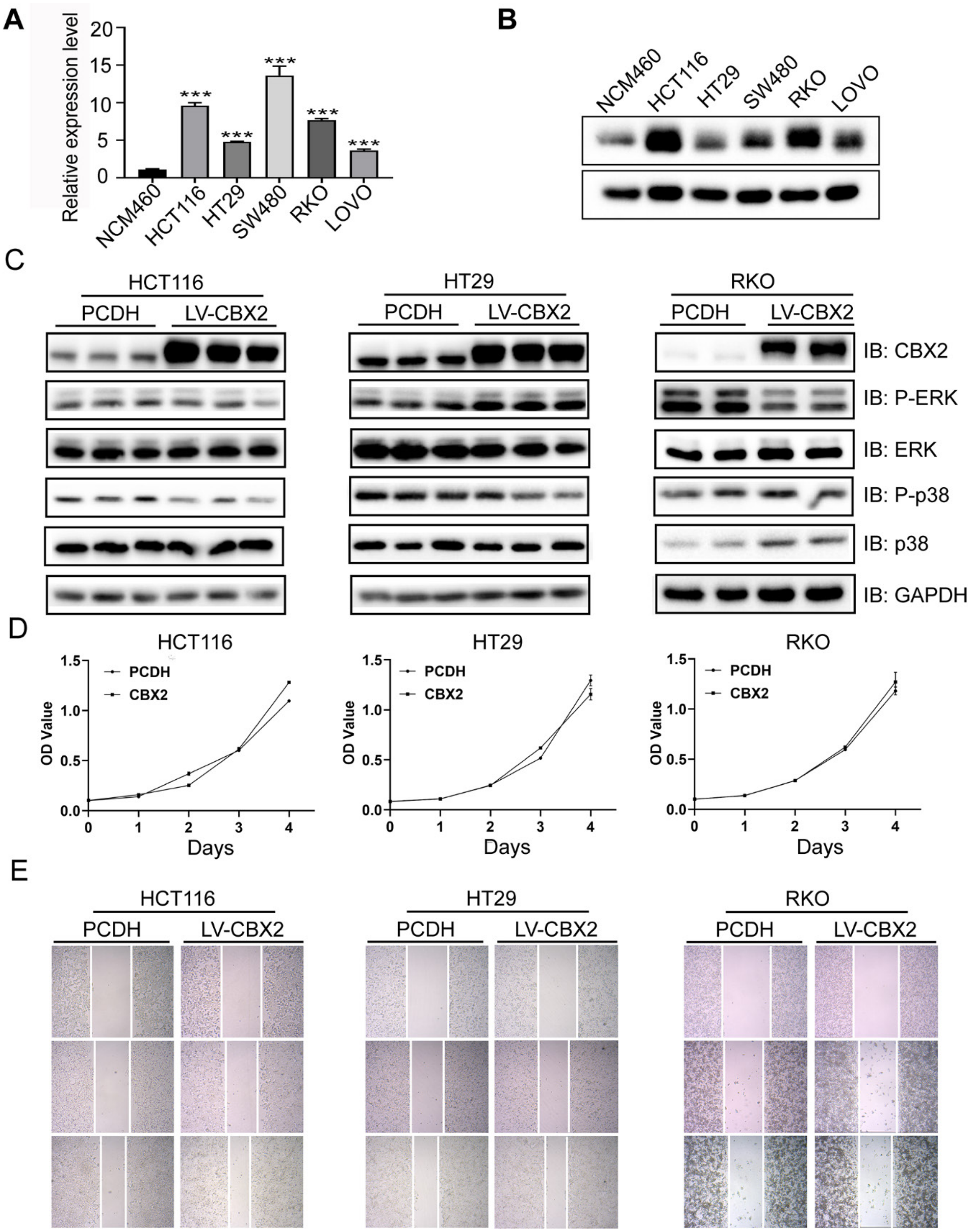

(A) The CBX2 mRNA level in CRC cell lines. (B) The CBX2 protein level in CRC cell lines. (C) WB of indicated proteins in CBX2 overexpression and control group. (D, E) MTT and wound healing assay are

performed to assess cellular proliferation and migration in CBX2 overexpression and control group.

Figure S2: The interaction of Mettl3 and CBX2

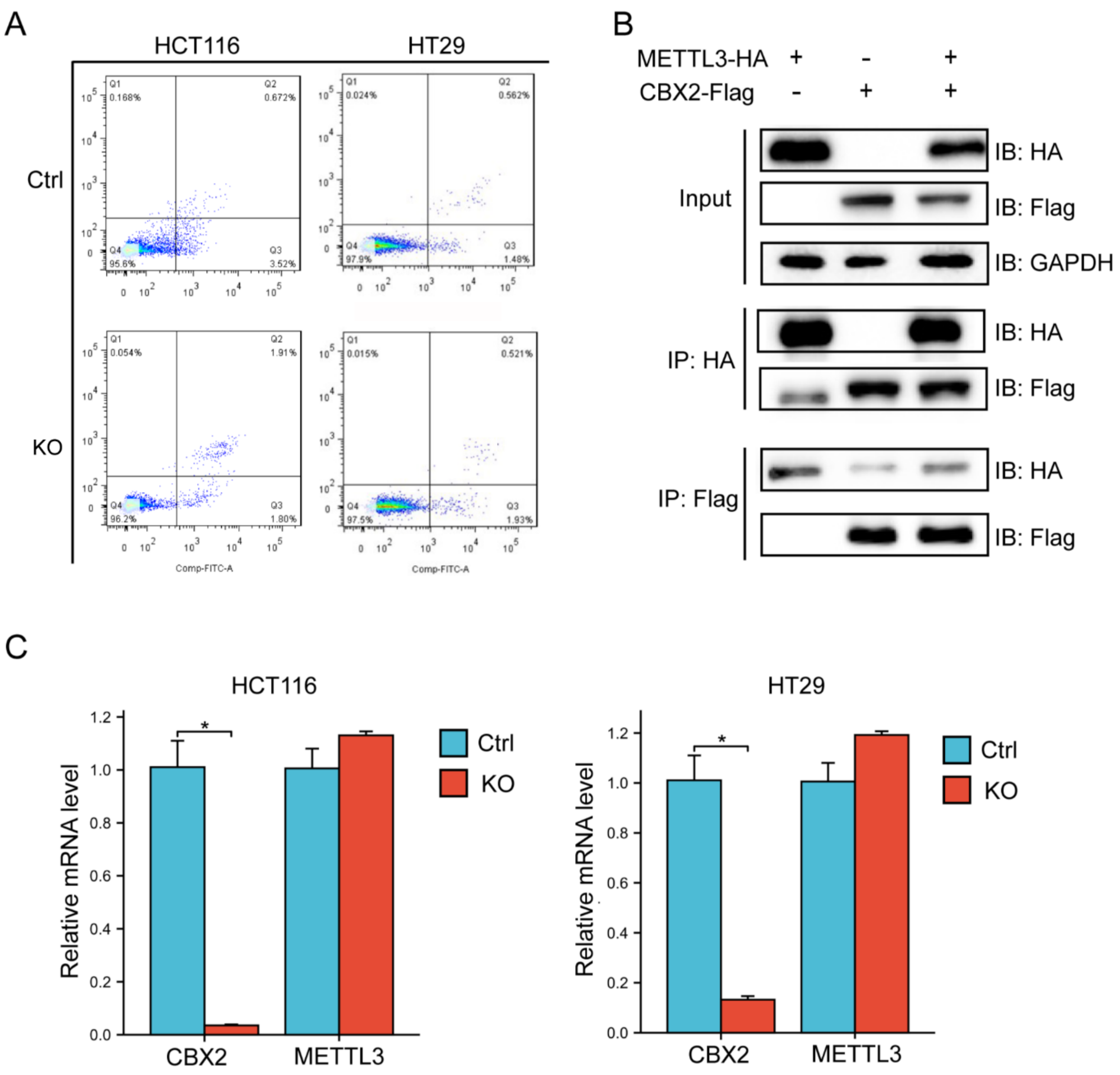

(A) Flow cytometry analysis is used to the detect the apoptosis in HCT116 and HT29 cells with or without CBX2 KO. (B) Co-IP is used to analysis the interaction of Mettl3 and CBX2. (C) qPCR is used to confirm the mRNA level of CBX2 and METTL3. Data are shown as mean ± SD; $*P < 0.05$.
